# Improved Electrical Signal of Non-Poled 3D Printed Zinc Oxide-Polyvinylidene Fluoride Nanocomposites

**DOI:** 10.3390/polym14204312

**Published:** 2022-10-13

**Authors:** Sharmad Joshi, Enrique Gazmin, Jayden Glover, Nathan Weeks, Fazeel Khan, Scott Iacono, Giancarlo Corti

**Affiliations:** 1 Department of Mechanical and Manufacturing Engineering, Miami University, Oxford, OH 45056, USA; 2 Department of Chemistry & Chemistry Research Center, United States Air Force Academy, Colorado Springs, CO 80840, USA

**Keywords:** zinc oxide, PVDF, 3D printing, cooling rates, poling

## Abstract

Polyvinylidene fluoride (PVDF) presents highly useful piezo and pyro electric properties but they are predicated upon the processing methods and the ensuing volume fraction of the *β*-phase. Production of PVDF with higher *β*-phase content for additive manufacturing (AM) is particularly desirable because it can enable the creation of custom parts with enhanced properties. Necessary steps from compounding to the testing of a 3D printed piezo sensitive sensor are presented in this paper. AM process variables and the influence of zinc oxide (ZnO) nanofiller on crystallinity, viscosity, and electromechanical properties of PVDF, have been explored. Fourier-transform infrared spectroscopy (FTIR) measurements confirm that a high cooling rate (HCR) of 30 °C min^−1^ promotes the conversion of the *α*-into the *β*-phase, reaching a maximum of 80% conversion with 7.5–12.5% ZnO content. These processing conditions increase the elastic modulus up to 40%, while maintaining the ultimate strength, ≈46 MPa. Furthermore, HCR 10% ZnO-PVDF produces four times higher volts per Newton when compared to low cooling rate, 5 °C min^−1^, pristine PVDF. A piezoelectric biomedical sensor application has been presented using HCR and ZnO nanofiller. This technique also reduces the need for post-poling which can reduce manufacturing time and cost.

## 1. Introduction

Polyvinylidene fluoride (PVDF) is a semi-crystalline polymer with a monomer unit of *CH*_2_–*CF*_2_ [1]. It has gained considerable attention in recent years due to its excellent piezoelectric and pyroelectric properties [2,3,4,5]. PVDF has a piezoelectric constant d33 of 20 × 10^−12^ CN^−1^ and a voltage constant g33 of 160 × 10^−3^ mVN^−1^ [6]. PVDF polymer shows less acoustic impedance and lower density compared to piezoelectric crystals [7,8]. PVDF has been used in sensors and actuators because it can convert mechanical deformations into coupled electric signals [4,9,10,11]. PVDF has also been employed in spin-valve devices because of its inherent properties such as thermal stability, short switching time, and large polarization. PVDF has also been used in capacitors, piezoelectric nanogenerators, [12,13,14,15,16], and nerve tissue engineering in biomedical applications [11]. From the five major crystalline polymorphs of PVDF, namely *α*, *β*, *γ*, *δ*, and *ϵ*, *β*-phase PVDF is of singular interest because the all-trans planar zigzag structure of molecules in this phase induces a net dipole moment that results in the aforementioned piezoelectric properties [17]. Thus, a variety of methods have been reported in the scientific literature to obtain *β*-phase PVDF. However, to the authors’ knowledge, there is minimal research available on mechanically compounding and 3D printing PVDF in the *β*-phase without poling.

Traditionally, the *α* to *β* transformation takes place when the *α*-phase is mechanically drawn or stretched [18] at temperatures less than 100 °C, with stretch ratios ranging from 1 to 5 [19]. Solvent casting techniques such as electrospinning and spin coating have been widely used to obtain thin-film composites or a raw material to fabricate filaments for Fused Deposition Manufacturing (FDM) [20,21,22,23,24,25]. A variety of nanofillers have also been used to nucleate *β*-phase crystals such as barium titanate, zinc oxide (ZnO) and titanium dioxide (TiO_2_) [26,27,28,29]. Certain PVDF copolymers such as P(VDF-TrFE) possess piezoelectric properties, as previously reported in the literature [23,30,31]. Dodds et al. found increased residual polarization with the addition of ZnO composites and speculated greater film piezoelectricity. Hu et al. also showed encouraging results demonstrating remnant polarization and *β*-phase crystallization with comparatively less amount of the nano-filler (graphene oxide or carbon nanotubes) [24]. Kim et al., Xiong et al., and Haddadi et al. used this process to obtain PVDF/silica composites where a marked transition from *α* to *β* phase was observed in all three cases, but a mixed result was seen in an attempt to enhance the mechanical properties of pure PVDF films [21,22,32].

Castanet et al. extruded a ZnO-PVDF nanocomposite and reported increments in *β* phase proportional to the amount of ZnO [27]. Kim et al. and Kennedy et al. employed a DMF solvent-cast approach to mix the nano-filler with PVDF before extruding the mix into FDM or Fused Filament Fabrication (FFF) or 3D print filaments. The 3D printed nanocomposite of Kim et al. used BaTiO_3_ as filler, and it was thermally poled after printing the samples [33,34]. Kennedy et al. used multiwalled-carbon nanotube (MWCNT) as a filler, and the 3D printed to be used as chemresistors rather than a piezoelectric sensor [35]. Porter et al. 3D printed PVDF films and demonstrated measurable piezoelectricity after a corona poling was applied [36]. Recently, a rapid precipitation 3D printing technique where a DMF-PVDF solution is 3D printed into a reservoir containing a non-solvent demonstrated a 78% improvement on the *β*-phase fraction after hot-pressing and poling the 3D printed samples [37]. Most of these procedures have only been utilized for obtaining thin films, limiting the capabilities of 3D printers, which is a flexible technique that can be employed to fabricate complex bulk specimens. Minimum research has been done on 3D printing bulk samples of piezoelectric plastics such as PVDF. Moreover, the few studies that have 3D printed PVDF composite thin films have also required the need of additional techniques such as thermal poling or solvent evaporation to obtain a higher concentration of *β*-phase PVDF or are limited to the mechanical and printability properties of PVDF [10,25,33,34,35].

**Figure 1:**
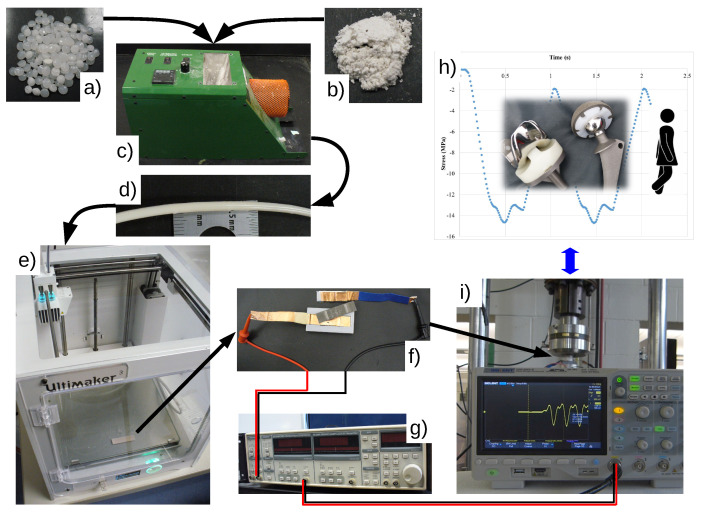
Materials, fabrication, and testing: (**a**) PVDF, (**b**) ZnO, (**c**) compounding and extrusion of the ZnO-PVDF nanocomposite into (**d**) 2.8 mm filaments were vacuum dried at 40 °C for 40 min, (**e**) 3D printing of test specimens, (**f**) electrically insulated test support with copper electrodes where the sample painted with conductive pain will rest, (**g**) lock-in amplifier connected directly to the test support electrodes, (**h**) walking profile with a 2 mm amplitude programmed into an MTS testing frame, and (**i**) measuring voltage generated during testing with an oscilloscope.

The research presented in this paper distinguishes itself from prior works by demonstrating how the flexibility of 3D printing can be harnessed to create PVDF parts directly in the *β*-phase. This effectively eliminates restrictions on sample/part shape and avoids time-cost intensive post processes, opening the possibility to 3D print sensors or bistable morphing generators [38]. Specifically, ZnO filler was used to promote *β*-phase PVDF formation in situ during the printing of the polymer composite material without poling. A simple and scalable manufacturing procedure (Figure 1) was envisioned by melt compounding PVDF and ZnO nanoparticles into a composite polymer filament to be used in commercially available FDM 3D printers. The extruded filaments were analyzed by differential scanning calorimetry (DSC), thermogravimetric analysis (TGA), Fourier transform infrared (FTIR) spectroscopy, and rheometry. Furthermore, this study expands on the effect that cooling rates have on the formation of the *β*-phase, without poling or stretching, to 3D print a ZnO-PVDF nanocomposite with an increased percentage of *β* phase content. Based on the material characterization results, two different 3D printing parameters were identified to directly affect the percentage of *β*-phase in the final printed specimen. Furthermore, the electrical and mechanical characterization methodology devised in this study demonstrates significant potential for creating 3D printed ZnO-PVDF sensors that are of custom design and could be integrated into shoe insoles to monitor human motion and posture. The various steps from the compounding of the ZnO-PVDF polymer to the creation and testing of the shoe insole sensor are illustrated in Figure 1 to explain the significance of the work from the materials and process design to application development.

## 2. Materials and Methods

### 2.1. Materials

PVDF composite filaments were extruded with PVDF from ARKEMA Kynar^®^ 740 with a molecular weight of 250,000 gmol^−1^ [3], melt flow rate ASTM D1238 of 1.5–3 kg, a density of 0.961 gcm^−3^, and melting point of 165–172 °C. Zinc oxide (30-1405, CAS 1314-13-2) with an average size of 20 nm and a density of 5.606 gcm^−3^ was purchased from Strem Chemicals. The conductive nickel paint (841AR Super Shield Nickel Conductive Coating) with a surface resistance of 0.7 Ωsq^−1^ for a thickness of 0.05 mm was purchased from MG Chemicals, Burlington, ON, Canada.

### 2.2. Extrusion

ZnO nanopowders were mixed in isopropyl alcohol and sonicated for 15 min, then PVDF pellets were added, and the mixture was sonicated for another 15 min. This mixture was placed in a vacuum oven at 40 °C to dry. The mixture was then compounded in an filament extruder (EX-2 from Filabot, Barre, VT, USA) with a 3 mm nozzle and placed 285 mm above the air path, which was set at 100% fan speed, to cool the extruded filament to ensure its roundness. The mixture was compounded for several passes to improve the uniform distribution of the ZnO nanoparticles throughout the whole filament. The first two passes were extruded at 170 °C at the maximum extruder speed and half speed of the spooler. Under these conditions, a 1.5 mm filament was achieved. The temperature was raised to 210 °C for the next three passes while maintaining the extruder and spooler speeds. In between each pass, the filament was mechanically pelletized and placed back into the extruder. The final pass was performed at an extruder temperature of 170 °C. The extruder and spooler speeds were decreased to ≈50% and ≈0.9 turns, respectively, to achieve a diameter of ≈2.5 mm required by the Ultimaker3 3D printer. At 170 °C, the extruder did not have enough torque to produce a uniform filament for 20% concentration of ZnO; thus the velocity of the extruder was reduced to accommodate this limitation.

### 2.3. 3D Printing

An Ultimaker3 3D printer (Ultimaker, Utrecht, The Netherlands) with a closed environment was used to print all samples. The mechanical properties of the PVDF nanocomposites were tested on four types of specimens. A 20 mm diameter by 1 mm thick rheometer specimens, 50 × 6 × 1 mm DMA specimen, ASTM D790 50.8 × 12.7 × 1.6 mm three-point bending specimen, and ASTM D638 Type V tensile specimen for tensile tests. 3D printer g-code was generated using Cura with 100% infill following the conditions shown in Table 1. Three samples of each specimen type were printed for both cooling profiles, High and Low Cooling Rates (HCR and LCR), and also for each zinc oxide percentage. The LCR profile was achieved by turning off the printer cooling fan, increasing the nozzle and bed temperatures, and maintaining a closed-door during the print. Due to the enclosed environment and the higher temperatures, there is a smaller temperature differential between the melted plastic and the environment. Instead, the HCR profile had a lower bed, and nozzle temperature, an open door, and the printer cooling fan was also on to maximum speed, thus forcing cooler room air on the printed specimen. Although PVDF does not absorbe water, all filaments were dried for 40 min in a vacuum dry oven at a 40 °C.

### 2.4. SEM Imaging and EDS

A field emission scanning electron microscope (FE-SEM) (Supra 35 VP FEG from Zeiss, Jena Germany) with an X-ray microanalysis system (XEDS) (Quantax 100 from Bruker, Billerica, MA, USA) was employed to analyze the fracture of the specimens. All specimens were coated with 5 nm of gold, and the images were taken at 5 KV to minimize charging with a working distance of 4 mm. Further characterization used mapping XEDS, and these spectra were collected using an accelerating voltage of 20 keV over a span of 300 live seconds at a 10k× magnification and 10 mm of working distance. The most prominent elements were fluorine (F), and zinc (Zn) which were used to identify the agglomeration of the ZnO nanoparticles.

### 2.5. Thermo-Gravimetric Analysis—TGA

The thermal stability of the ZnO-PVDF and PVDF-TiO_2_ composite and the filler concentration were measured via a thermogravimetric analyzer (TGA) (TGAQ500 from TA Instruments, New Castle, DE, USA). All samples were heated in a nitrogen (N_2_) atmosphere up to 850 °C at a heating rate of 10 °C min^−1^. Three random samples from the ends and the middle section of each filament were tested and analyzed using the TA Universal Analysis software (TA Instruments, New Castle, DE, USA).

### 2.6. Differential Scanning Calorimetry—DSC

Differential scanning calorimetry (DSC) was employed to identify the melting ($T_{m}$), crystallization ($T_{c}$), and glass transition ($T_{g}$) temperature of the samples. Two cooling rates were tested to identify the cooling rate’s effect on the crystallization of PVDF nanocomposites, a LCR of 5 °C min^−1^, and a HCR of 30 °C min^−1^. $T_{m}$ was always measured at a heating rate of 5 °C min^−1^. Considering that higher heating rates provide a better resolution to identify the $T_{g}$ point, a 10 °C min^−1^ was selected for these experiments. All DSC data were collected on the third run. Three samples from the ends of each filament were characterized using DSC TAQ100 (TA Instruments, New Castle, DE, USA). The collected data were analyzed with TA Universal Analysis software (TA Instruments, New Castle, DE, USA).

### 2.7. Fourier Transform Infrared Spectroscopy—FTIR

The polymeric nanocomposite material was 3D printed into specimens following the printing procedure stated earlier. The samples were characterized using a Nicolet™ iS™ 5 FTIR Spectrometer (Thermo Fisher Scientific, Waltham, MA, USA). FTIR measurements were collected at one cm^−1^ with 32 scans. All DSC samples and all printed DMA specimens were analyzed by FTIR.

### 2.8. Rheometry

The rheology data was collected on a HAAKE RheoWin 4.82.00 with a MARS 40 measuring device (Thermo Fisher Scientific, Waltham, MA, USA). A PP20 Adapter-01180765 was used for measuring the geometry. The PDVF-ZnO samples in Table S1 were all analyzed on the rheometer at three temperatures, 200 °C, 225 °C and 250 °C. The instrument was heated to the target temperature and then the sample was loaded into the test chamber. The instrument then allowed the sample to heat up for 120 s. Following the temperature adjustment, the instrument then began collecting data and ran for 100 s. During the 100 s^−1^ test interval, the instrument measured steady viscosity (Pa·s) vs. the shear rate (s^−1^).

### 2.9. DMA

The width and thicknesses of all DMA specimens were measured at three different points, and the average dimension was used in the stress and strain calculations. A RS3 DMA (TA Instruments, New Castle, DE, USA) was used to measure the storage modulus (E’), loss modulus (E”), and tan(δ) (E”/E’). During testing, specimens were allowed to stabilize at room temperature of 23 °C by introducing an isothermal segment of 2 min before a ramp-up cycle up to 150 °C at a rate of 5 °C min^−1^. Data were collected at an interval of 10 s.

### 2.10. Tensile and Flexion

Tensile tests were conducted according to the ASTM D638 standard type V for tensile properties of reinforced plastics using [39]. Similar to the DMA test, the widths and thicknesses of all the samples were measured at three different points, and the average of these dimensions was used for the calculation of tensile strength and modulus of the specimen. The crosshead speed was set at 1 mm·mm^−1^·min^−1^ according to the standard [39]. The specimen was allowed to reach the rupture point. The load and the extension and of each specimen were recorded every 50 ms. Three samples for each printing condition and material combination were tested.

Flexural tests were conducted according to the ASTM D790 standard. In this case, a 3 point bend test fixture was fitted in the grippers of 30 kN Instron. Subsequently, the specimen was tested by placing them centrally on the supports. The crosshead speed was set at 1 mm·min^−1^ according to the standard [40]. Similar to the tensile test, the dimensions were taken at three different points and the average was used for the calculation of flexural modulus. The load and the extension of each specimen were recorded every 20 ms. Three samples for each printing condition and material combination were tested. Both tensile and flexural tests were performed on a Universal test frame (series 5960 Instron, Norwood, MA, USA).

### 2.11. Electrical Response

The electrical response of the 3D printed PVDF and ZnO-PVDF nanocomposite was measure in a three-point bending test under a cyclic strain of 0.2–0.4% at 6 Hz initially and later at 0.8 and 4 Hz to match the walking gait profile of an average person [41]. The ASTM D790 3-point bending specimens were coated with a conductive nickel paint with an approximate thickness of 0.5 mm. The RS3 DMA (TA Instruments, New Castle, DE, USA) was employed to strain the sample for 10 s. A 3-point bending plastic support with a 25 mm span (Figure 1) fitted with copper electrodes was mounted on the DMA. The copper electrodes were connected with a BNC cable to a lock-in amplifier (SR810 from Stanford Research Systems, Sunnyvale, CA, USA) and the output signal was measured with an oscilloscope (SDS1104X-E from Siglent, Shenzhen China). The applied force was calculated using the measured dimensions and the average modulus of elasticity of the samples. The simulated walking gait profile was programmed into a hydraulic MTS load frame (100 kN Landmark from MTS, Eden Prairie, MN, USA) and use the same electrical connections as previous tests. Two samples samples for each printing condition and material combination were tested.

## 3. Results and Discussion

During the filament extrusion provides the first insights on the behavior concentration of ZnO nanopowder.

### 3.1. Materials Characterization

**Figure 2:**
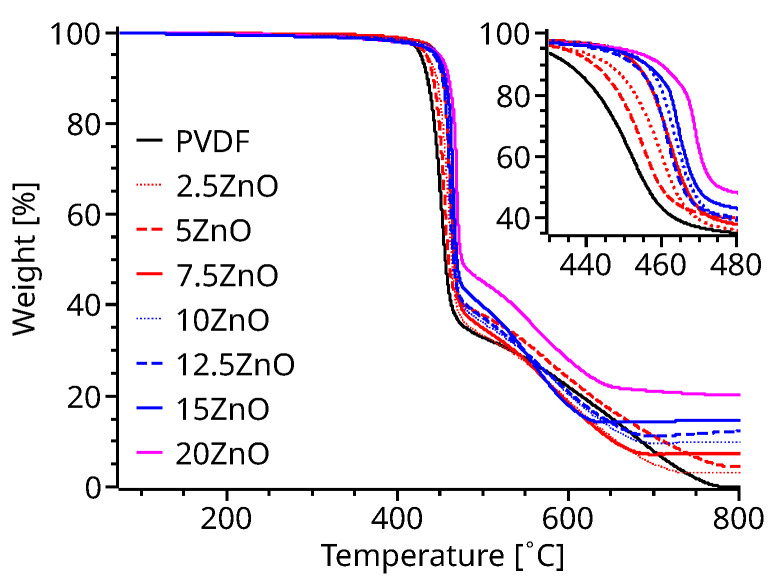
TGA measurements (inset shows a magnification in the area of interest).

Since 3D printing PVDF requires a nozzle temperature of 250 °C and PVDF decomposes in toxic fumes, the thermal stability of the PVDF nanocomposite was first evaluated. Thermogravimetric analysis of ZnO-PVDF (Figure 2) shows a thermal stability increase as a function of the ZnO content. The decomposition temperature increases up to 25 °C higher than that of pristine PVDF for a 20% content of ZnO. This increased thermal stability is attributed to the interaction between the polymer and ZnO nanoparticles. TGA data also determined the amount of ZnO present in each compound filament. Table S1 shows the average amount of ZnO remaining after the calcination of PVDF, while there was a loss of ZnO during compounding, TGA measurements show a low standard deviation and error on the ZnO weight left after PVDF’s calcination, suggesting a uniform extruded filament.

The glass transition temperature (Figure S1), measured using a differential scanning calorimetry, increased as a function of the percentage of ZnO content of the samples. In agreement with existing literature, smaller changes were noticed for samples with less than 7.5% of ZnO, which presented T${}_{g}$ at −40.1 °C and 30.9 °C for pristine PVDF and 7.5% of ZnO, respectively. 20% ZnO samples showed the highest T${}_{g}$ at −7.5 °C (Figure S1). The increasing T${}_{g}$ can be explained by the lower molecular mobility of the PVDF chains induced by the ZnO nanoparticles’ and the crystallinity of ZnO [42].

**Figure 3:**
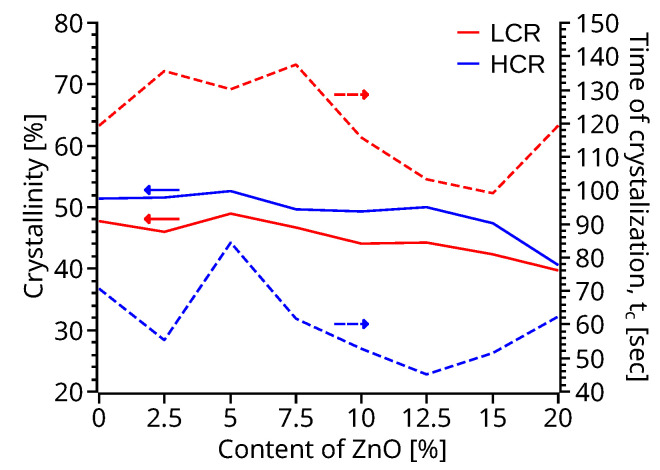
Comparison of the crystallinity percentage and the time of crystallization, $t_{c}$ as function of the ZnO sample content and cooling rate.

The crystallinity of PVDF and ZnO-PVDF nanocomposite samples (Figure 3) was calculated using Equation (1) with a melting enthalpy of 100% PVDF $H_{f}^{*}$ equal to 104.6 Jg^−1^, and heat flow $\Delta H_{f}$ equal to the area under the crystallization peak (Figure S2) [43,44,45]. The overall crystallization time (Figure 3) was calculated by subtracting the time at the crystallization peak temperature from the on-set temperature-time (Figure S2). The effect of the cooling rate on the crystallinity and time of crystallization was analyzed by cooling the samples at two different rates: a LCR of 5 °C min^−1^ and a HCR of 30 °C min^−1^.

(1) $$X_{c}=\frac{\Delta H_{f}}{\Delta H_{f}^{*}}\times 100\%.$$

High cooling rate samples produced a slightly larger percentage of crystallization (Figure 3) than the low cooling rate samples, while with HCR, the degree of crystallization is relatively stable until 15% of ZnO; thereafter, the crystallinity decreases. This behavior is also observable on LCR, where the crystallinity of the samples drops for contents of ZnO larger than 12.5%. This reduction of crystallinity could be attributed to the reduction of nucleation sites due to the agglomeration of the ZnO nanoparticles as shown on the mapping XEDS (Figure S3) due to the increased content of ZnO as previously reported by Castanet et al. [27]. ZnO nanoparticles have a significant contribution, mainly in the range of 10–15% of ZnO, on the reduction of the time to crystallization (Figure S1), ≈35% and ≈12% for the HCR and LCR, respectively, with respect to pristine PVDF. The reduction of the time to crystallization can be attributed to the increased nucleation sites created by the presence of the ZnO nanoparticles. The ZnO nanofiller also increased the temperature of crystallization (Figure S2), reaching up to ≈7 °C higher for both cooling rates. Samples with 10–15% ZnO also depict a narrower crystalization peak compared to pristine PVDF, suggesting a narrow crystallite size distribution [46,47].

**Figure 4:**
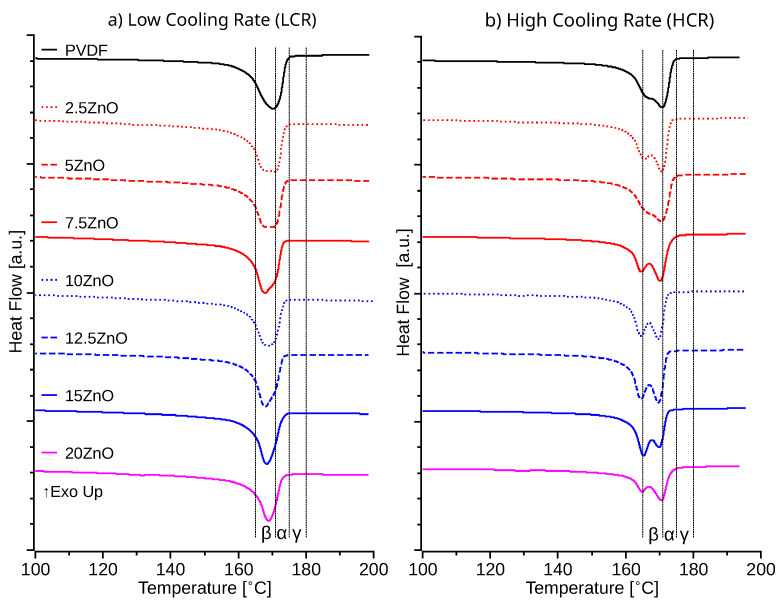
DSC melting temperature for (**a**) 5 °C min^−1^ LCR, and (**b**) 30 °C min^−1^ HCR.

Following the crystallization cycles, both high and low cooling rate samples were melted at 5 °C min^−1^. DSC melting cycles following LCR exhibited a lower temperature shoulder or a decrease of the melting temperature, with respect to pristine PVDF, 170.3 °C, for all ZnO-PVDF samples above 5% of ZnO (Figure 4). Samples with 7.5% of ZnO presented the lowest melting temperature at 167.9 °C for LCR samples. Instead, HCR produced a double melting peak or a shoulder of the main melting temperature peak for all samples. This lower-temperature melting peak decreased with the increase of ZnO content until reaching a minimum with 15% of ZnO content at 164.4 °C, which is the melting temperature of *β*-phase of PVDF. Sencadas et al., Harstad et al. and Soin et al. reported the formation of a shoulder and a second peak at 165 °C to be proportional to the increment the PVDF *β*-phase [48,49,50]. Literature also indicates that the *γ*-phase have a 175 °C < *T_m_* < 175 °C, the *α*-phase have a 172 °C < *T_m_* < 180 °C, and the *β*-phase has a 165 °C < *T_m_* < 172 °C [12,14,16,50,51]. Both HCR and LCR samples, present no visisble peaks or shoulders at temperatures above 175 °C, suggesting a minimum presence of the *γ* phase on the ZnO-PVDF nanocomposite [51].

FTIR spectra (Figure S4) were collected from the bottom of the DSC samples after cooling the samples at HCR and LCR on the third DSC cycle. There are no appreciable shoulders on 1234 cm^−1^, suggesting the lack or minimum content of the *γ*-phase. The 840 cm^−1^, which represents the *β* phase and the *γ*-phase, decreases its transmittance as a function of the ZnO reaching a minimum transmission with 10–12.5% of ZnO, for both HCR and LCR samples. Similarly, the 1275 cm^−1^ peak, which represents the *β* phase solely, decreases its transmittance as a function of the amount of ZnO reaching a minimum transmission with 10% of ZnO, while the *α* phase 760 cm^−1^ increase for both HCR and LCR samples. These findings are in agreement with existing literature [14,37,51,52]. Moreover, FTIR result corroborate the findings from DSC melting temperatures. Based on these findings, two FDM or 3D printing profiles were designed, Table 1, to mimic as close as possible both DSC, high and low, cooling rates. It is important to mention that the 3D printed LCR profile was limited by the 3D printing environmental control, which produced a cooling rate higher than 5 °C·min^−1^. Specimens for rheometry, DMA, tensile and flexural tests were 3D printed for subsequent analysis of the PVDF and ZnO-PVDF nanocomposite mechanical properties.

**Figure 5:**
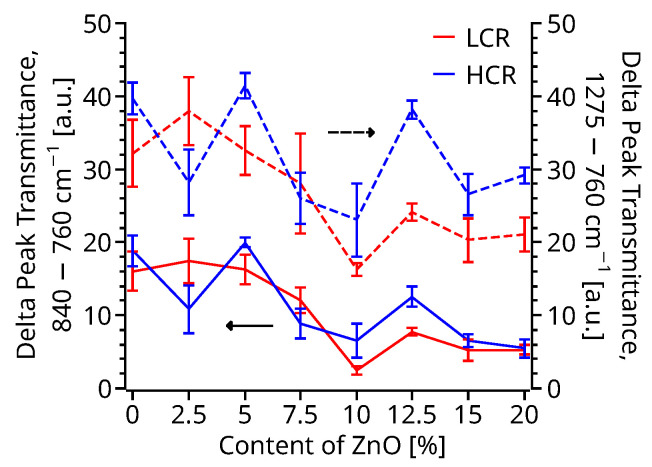
Comparison of transmittance changes for the *α* and *β* peaks as a function of the ZnO sample content and cooling rate.

FTIR measurements (Figure S5) were also performed on the upper surface of all the 3D printed DMA specimens. These spectra exhibit a reduction of the transmittance on the 400–500 cm^−1^ band assigned to the characteristic stretching mode of Zn-O bond proportional to the ZnO percentage in the sample. In addition (Figure 5), the α peak, 760 cm^−1^, increases its transmittance as a function of the ZnO percentage, reaching a maximum at 10–12.5% of ZnO, while the *β* − *γ* peak, 840 cm^−1^, and the *β* peak, 1275 cm^−1^, decrease their transmittance proportionally to the percentage of ZnO. A minimum transmittance for the peak of the 840 cm^−1^, and 1275 cm^−1^ peaks was found at concentrations of 10–12.5% of ZnO. Similarly to the DSC samples, there is no apparent change to the 1234 cm^−1^ *γ* peak. When comparing the changes in transmittance, there is also a reduction proportional to the ZnO percentage with a maximum of 80% reduction (Figure 5) of the *α* minus *β* ratio with respect to the pristine PVDF for the 10% ZnO samples. Due to the smaller difference in cooling rates of the 3D printed samples compared to the DSC samples, there are no statistical differences between the two printing profiles.

### 3.2. Rheological and Mechanical Properties

**Figure 6:**
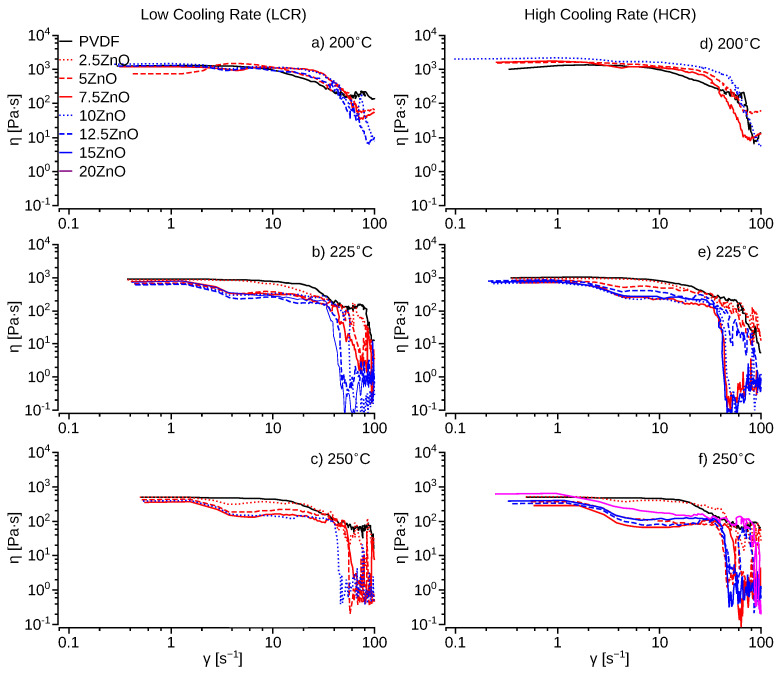
Shear viscosity *η* versus shear rate *γ* at different temperature profiles for low and high cooling rate 3D printed samples.

Lower shear viscosity and elasticity have been related to ease of 3D printing and quality [53,54]. The viscosity curves (Figure 6) obtained from the rheometer show a broad plateau of constant shear viscosity, e.g., below shear rates of 10 s^−1^ for pristine PVDF. Above shear rates of 10 s^−1^, there is a significant reduction, e.g., one order of magnitude, of the shear viscosity as a function of the temperature. There are no significant differences between the HCR and LCR samples using pristine PVDF. However, ZnO-PVDF nanocomposite samples exhibit increased shear stress (Supporting Information Figure S5) and shear viscosity (Figure 6) at temperatures below 200 °C. Suggesting, a reinforcing effect due to the ZnO nanoparticles. This effect also increased pressure on the extruder for ZnO concentration above 15%, resulting in a reduction of the extrusion speed to produce a uniform filament. However, at higher temperatures, above 225 °C, ZnO produced a reduction of the shear viscosity of ≈678 and ≈827 Pa·s for the LCR and HCR, respectively, at lower shear rates of ≈2 s^−1^ than the pristine PVDF. The thinning effect of the ZnO is more significant with HCR of PVDF nanocomposites, reaching a minimum shear viscosity of ≈68 Pa·s, and shear stress of ≈680 Pa, at 250 °C and 10 s^−1^, for a ZnO concentration of 7.5–12.5%. The ZnO thinning effect can be attributed to the ZnO nanoparticle size being smaller than the polymer’s RMS radius of gyration as seen before by Mackay et al. and Kairn et al. [55,56]. However, as the concentration of ZnO increases above 12.5%, the shear viscosity (Figure 6) and shear stress of PVDF (Figure S6) began to increase. The filler-like behavior is attributed to the large concentration of ZnO and agglomeration of the nanoparticles (Supporting Information Figure S3). Similar thinning behavior, for <12.5% ZnO, and filler-like behavior, for >12.5% ZnO, is present in both the LCR and HCR samples for temperatures above 225 °C. Due to the thinning effect of ZnO, PVDF-ZnO nanocomposites have significant lower viscosities, ≈10 Pa·s, than pristine PVDF, ≈100 Pa·s, at higher shear rates, e.g., ≥100 s^−1^. Thus, making PVDF-ZnO nanocomposite a better option for FDM 3D printers since typical shear rates at the nozzle are in the range of 10^2^–10^3^ s^−1^ depending on the printing filament speeds [54].

**Figure 7:**
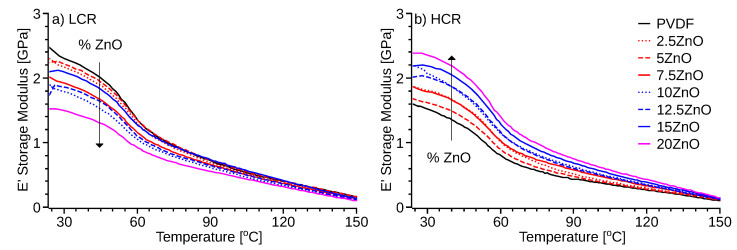
Storage modulus (E’) of DMA specimens 3D printed with: (**a**) low cooling rate (LCR), and (**b**) high cooling rate (HCR).

DMA specimens of both 3D printing profiles show a storage modulus (Figure 7), an order of magnitude higher than the loss modulus (Figure S7). Hence, material elasticity remains dominant at lower temperatures. In the high-temperature region, the difference in the magnitudes of the two moduli reduces to an extent, yet the loss modulus remains lower than the storage modulus. In the LCR samples, E’ (Figure 7a) and E” (Figure S7a) decrease with the increasing percentage of ZnO. A similar trend was observed in the material viscosity (Figure 6). The HCR specimens show a proportional elastic modulus (Figure 7b) and loss modulus (Figure S7b) to the percentage of ZnO. The stiffening effect could be due to the lower time to crystallization (Figure 3), good dispersion of the nanoparticles in the matrix, and the high surface area of the ZnO particles as reported by Cao et al. [57]. The increment in the storage modulus (Figure 7b) indicates that HCR condition with ZnO-PVDF nanocomposite material creates higher resilience to high temperatures. The opposite trend observed in LCR specimens (Figure 7a) indicates that lower cooling rates hinder the stiffening effect of ZnO nanoparticles. Similar results were obtained by Flyagina et al. [58]. The larger crystallites may explain the softening effect that slow cooling has on ZnO-PVDF nanocomposite due to the longer time to crystallization of ZnO-PVDF nanocomposite (Figure 3).

**Figure 8:**
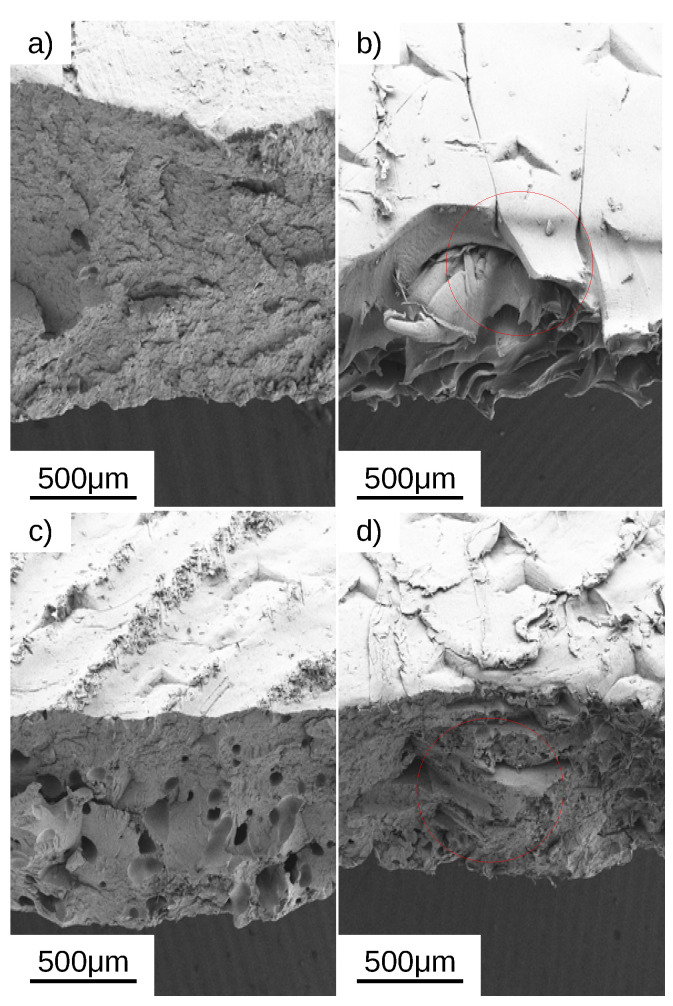
SEM micrographs of representative tensile specimens 3D printed with a [45°, 135°] infill direction: (**a**) LCR pristine PVDF, (**b**) HCR pristine PVDF, (**c**) LCR of 10% ZnO-PVDF, and (**d**) HCR of 10% ZnO-PVDF. The red circles shows elongation characteristic of the ductile failure of the HCR sample.

**Figure 9:**
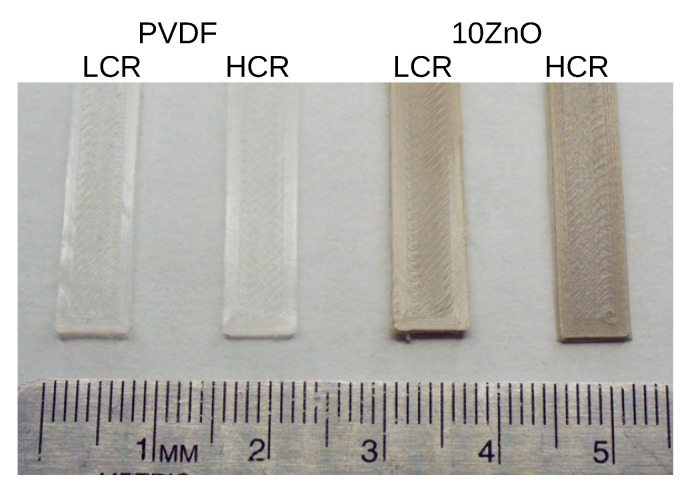
Optical image comparing DMA specimens 3D printed with a [45°, 135°] with HCR and LCR printing profiles.

Tensile and flexural tests were also carried out following ASTM D638 and D790 for both cooling rates specimens [39,40]. Two infill configurations were studied; an infill orientation [0°, 0°], and an infill orientation of [45°, 135°] with respect of the tensile force. Pristine PVDF has a natural elongation, which decreases with an increased percentage of ZnO filler (Supporting Information Figure S8a,b) when printed at LCR regardless of the printing infill direction. HCR printed samples produced a higher elongation than LCR for pristine PVDF at low concentrations of ZnO, e.g., <5%, (Supporting Information Figure S8c,d) regardless of the infill printing orientation. SEM images of the tested specimens also corroborate these findings. LCR presented minimum deformation at the breaking point, while HCR showed (Figure 8) a plastic deformation before rupture. PVDF and PVDF-ZnO nanocomposite produced high-quality prints (Figure 9) with no appreciable difference between HCR and LCR printing profiles.

**Figure 10:**
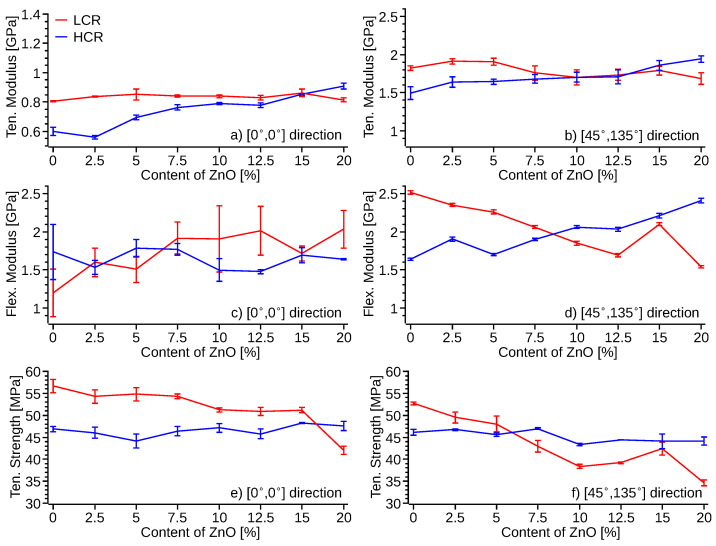
Average tensile and flexural test results of specimens 3D printed with HCR (blue) and LCR (red). Elastic modulus from tensile test (**a**) [0°, 0°] infill direction, (**b**) [45°, 135°] infill direction, flexural modulus from three-point bending (**c**) [0°, 0°] infill direction, (**d**) [45°, 135°] infill direction, tensile strength of 3D printed specimens with (**e**) [0°, 0°] infill direction, and (**f**) [45°, 135°] infill direction.

Further analysis of the tensile specimens showed an increase of ≈25% in the elastic modulus (Figure 10b,d) between pristine PVDF and the 20% of ZnO HCR sample with a [45°, 135°] infill. The modulus for specimens printed with [0°, 0°] infill orientation and HCR (Figure 10a,c) also increases proportionally to the percentage ZnO in the samples, to a maximum of 0.9 GPa, approximately 30% larger than the modulus of pristine PVDF. The lower modulus of the [0°, 0°] infill direction samples is attributed to a higher degree of anisotropy and defects of the [0°, 0°] printed samples. Three-point flexion tests of the HCR samples also display a similar increase of the elastic modulus (Figure 10c,d) with the increasing percentage of ZnO nanofiller, with a maximum elastic modulus of 2.0–2.3 GPa, which is ≈40% greater than that of pristine PVDF. The elastic modulus of the LCR samples printed with [0°, 0°] infill direction shows no statistically significant difference between the pristine PVDF and ZnO-PVDF nanocomposite specimens. The elastic modulus of LCR samples printed with a [45°, 135°] infill pattern decreased as the content of ZnO is increased in the ZnO-PVDF nanocomposite. 3D printed specimens, regardless of the infill direction, with HCR produced a significant increase in the elastic modulus (Figure 10b,d) as a function of the ZnO content. The elastic modulus increase could be attributed to the lower time to crystallization (Figure 3), increased *β* phase formation (Figure 5), and the stiffening due to the nanofiller as reported previously in the literature [27,59].

The strength of the 3D printed specimens (Figure 10e,f) with HCR did not show any statistically significant difference between pristine PVDF samples and ZnO-PVDF nanocomposite for either of the infill directions [0°, 0°] and [45°, 135°]. LCR produced stronger pristine PVDF specimens, but the strength reduces with increased content of ZnO. These results were confirmed by testing the filament used to print the specimens (Figure S9). The extruded filament was cooling down to room temperature using fans. Thus, its cooling rate is comparable to the HCR rate of the 3D printed samples. The filament displayed no statistical difference in strength, except for the 20% of ZnO. The filament composite displayed an increase of the elastic modulus proportional to the content of ZnO with a maximum elastic modulus of ≈2.1 GPa, an increase of 30% with respect to the pristine PVDF. This result is in agreement with the strength and elastic modulus of the specimens 3D printed with HCR and a [45°, 135°] infill pattern.

### 3.3. Electrical Response

**Figure 11:**
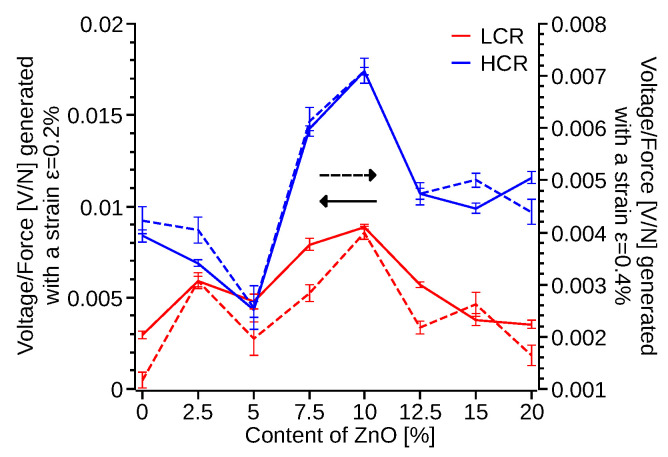
Average volts per newton generated by the 3D printed PVDF and ZnO-PVDF nanocomposite samples as function of the percentage of ZnO for low (red) and high (blue) cooling rates.

The electrical response (Video S1) of the 3D printed specimens was also measured. The three DMA samples, with infill direction of [45°, 135°], were coated with conductive nickel paint with an averageresistance of 2.2 ± 0.2 Ω at 25 mm. These samples were subjected to 6 Hz cyclic loading, a 0.2% and 0.4% strain amplitude with a span of 25 mm. When the locking amplifier was set to match the driven frequency of the DMA (Figures S10 and S11), the signal saturate at the peak voltage, reaching a maximum after two seconds of oscillating input. The driving force of the DMA was calculated as a function of the measured elastic modulus of the samples (Figure 10d) and the input strain. The measured voltage was related to the force and plotted as a function of the content of ZnO (Figure 11). Samples with 7.5–15% ZnO produced a two fold increment of the voltage per unit force with respect of the pristine PVDF samples. Furthermore, the HCR samples produced ≈70% increment of the generated voltage compare to the LCR and approximately a four times higher voltage per unit force (Figure 11) for HCR 10% ZnO-PVDF with respect of LCR pristine PVDF. These results correlate with the FTIR (Figure 5) and DSC (Figure 4) measurements, which show that samples with 7.5–15% ZnO had a larger percentage of *β* phase. ZnO nanoparticles may have also improve the charge motion to the electrodes, in accordance with Zhang et al. findings, and thus increasing the voltage signal [60].

**Figure 12:**
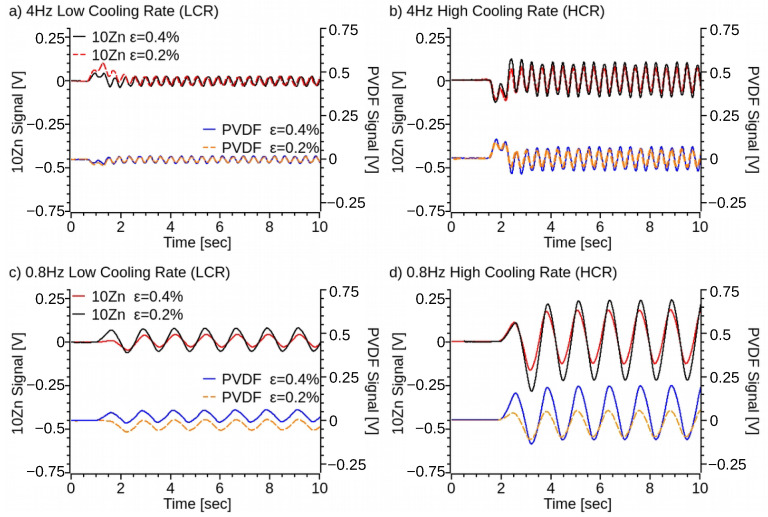
Voltage signal generated by the 3D printed PVDF and ZnO-PVDF in response to a 0.2% and 0.4% sinusoidal strain at a frequency of: (**a**) 4 Hz for LCR samples, (**b**) 4 Hz for HCR samples, (**c**) 0.8 Hz for LCR samples, and (**d**) 0.8 Hz for HCR samples.

Experimental testing also revealed a frequency dependency of the signal generated by the test samples. As the setting frequency on the locking amplifier increased above the driven frequency, the signal of the PVDF-nano composite samples became sinusoidal and its frequency increased proportional to the locking amplifier’s frequency set point. When the locking amplifier matching frequency is set to two times the driven frequency, then the specimen signal match (Figure 12a,b) the input force frequency. Setting the locking amplifier at a higher matching frequency increases the signal frequency and decreases the signal amplitude (Figure 12c,d), while the frequency response remains constant, ZnO increases (Figure 11) the signal amplitude, with 10% of ZnO content producing the maximum volts per newton of applied force, ≈0.0175 V/N. Similarly, HCR samples (Figure 11 and Figure 12) increase the voltage to force signal by approximately two times regardless of the ZnO content, with the exception of the 5% of ZnO. 5ZnO also presents the lowest (Figure 5) alpha to beta transmittance difference, and its melting point (Figure 4) is similar to pristine PVDF, suggesting a lower percentage of *β*-phase formation. This effect is attributed to the higher concentration and agglomeration of ZnO (Figure S3).

**Figure 13:**
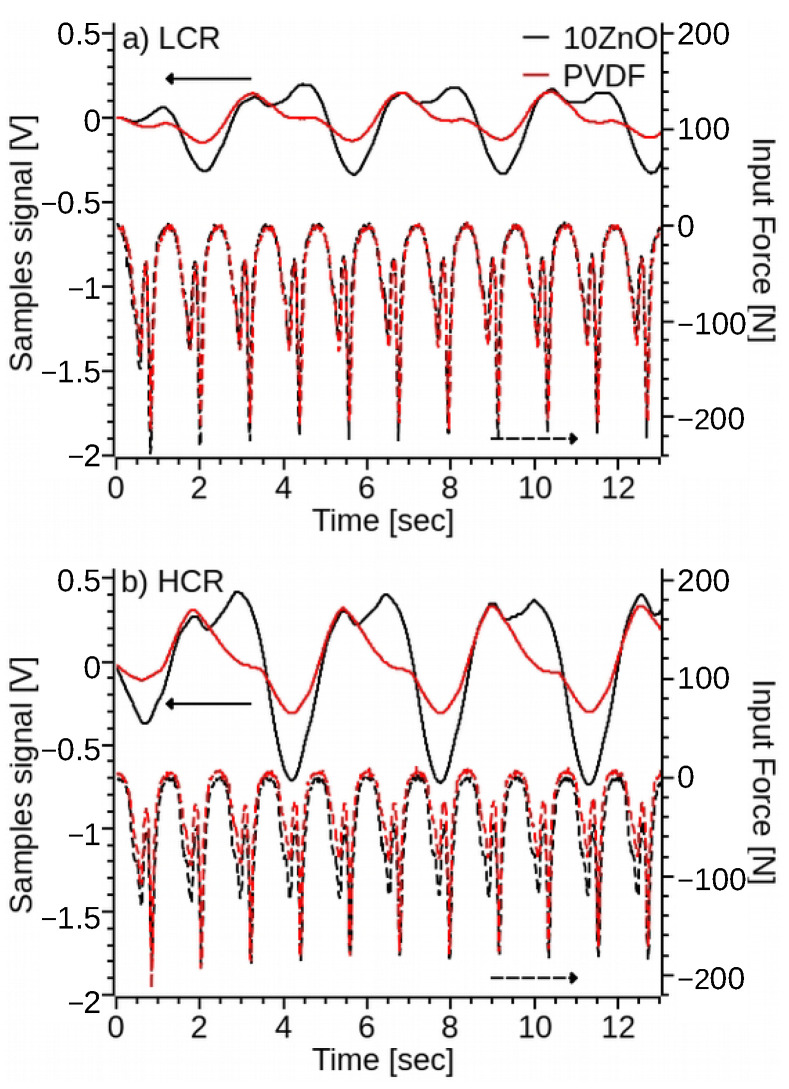
Voltage signal generated in response to a walking profile simulated on an MTS tensile frame for by pristine PVDF and 10%ZnO PVDF nanocomposite samples created with: (**a**) LCR and (**b**) HCR 3D printing profiles.

Since the 10ZnO specimens produce a better electrical response, they were also tested by applying a strain that simulates a walking profile (Figure 13) of an average person (Video S2) [41,61]. The walking profile was set to a maximum deflection amplitude of 2 mm, while the MTS test frame measured the force, and the sample signal was amplified and measured with the same setup from previous experiments. The 10Zn samples, HCR and LCR, were capable of reproducing the input signal (Figure 13) with higher accuracy than the signal of pristine PVDF samples (Video S2). Similar to the frequency tests on the DMA samples (Figure 12) and due to the superimposed frequencies of the walking profile, there is a frequency mismatch with respect to the applied wave. The HCR specimens for the PVDF and 10Zn specimens produced ≈2 times more volts per newton compared to the LCR specimens. The 10Zn also produced a ≈30% and ≈20% increase in V/N than pristine PVDF samples for HCR and LCR specimens, respectively. The 10Zn samples, HCR and LCR, were capable of reproducing the input signal (Figure 13) with higher accuracy than the pristine PVDF samples (Video S2). The increased signal of the 10Zn samples with respect to pristine PVDF is similar to the observed V/N at a constant driving frequency (Figure 12). Two 10ZnO samples were tested (Figure S12) under the same condition producing an equivalent electrical response to the applied load. Similarly, the two PVDF specimens produced an equivalent response. These results suggest that the 3D printed ZnO-PVDF composite material is repeatable and correlates to the locking amplifier’s matching frequency. Thus, the need for a frequency response characterization of the sensor, which is outside of the scope of the current project.

## 4. Conclusions

PVDF and PVDF metal oxide nanocomposites were compounded and extruded into filaments for FDM or FFF 3D printers. The filaments properties were characterized by analyzing thermal stability, crystallinity, and crystal phase composition by using TGA, DSC, and FTIR. Furthermore, the filaments’ viscosity was studied at temperatures close to the nozzle temperature to 3D print PVDF. The mechanical properties of 3D printed parts were also characterized along with the electrical output to a mechanical strain. TGA analyses exhibited an increase of ≈25 °C of the decomposition temperature with respect to pristine PVDF. DSC measurements also showed an increase in the glass transition temperature as a function of the ZnO content. Although DSC did not show a significant increase in crystallinity of the nanocomposite filament as a function of the ZnO content, it did exhibit a ≈35% and ≈12% reduction in the time of crystallinity for a HCR of 30 °C min^−1^ and LCR of 5 °C min^−1^, respectively, for samples with a 12.5–15% ZnO. Samples with 10–15% of ZnO also depict a narrower crystalization peak compared to pristine PVDF, suggesting a narrow crystallite size distribution. DSC melting cycles following a HCR of 30 °C min^−1^ produced a double melting peak. The lower temperature melting peak increase with the ZnO content until reaching a minimum with 15% at 164.4 °C, which is typically attributed to PVDF *β*-phase. FTIR corroborated the DSC measurements and showed a decrease of the 840 cm^−1^ and 1275 cm^−1^ peaks, which represent the *β* phase, reaching a minimum transmission with 10–12.5% of ZnO content. The *α*-phase 760 cm^−1^ increased as a function of the amount of ZnO, reaching an 80% reduction of the *α*/*β* ratio with 10–12.5% of ZnO. The ZnO-PVDF nanocomposite filament’s shear viscosity reduced as a function of the ZnO percentage reaching a minimum at 7.5–12.5% of ZnO, due to the size of the ZnO nanoparticles. Overall the HCR ZnO-PVDF produces up to ≈40% increase of the elastic modulus while maintaining the same ultimate strength of the pristine PVDF. Furthermore, concentrations of ZnO up to 5% increased the toughness of the ZnO-PVDF nanocomposite. Printing infill direction does not affect HCR samples, but it increases the PVDF and ZnO-PVDF nanocomposite strength when 3D printed with an LCR. FTIR measurements indicate that 7.5–12.5% of ZnO promote up to 80% conversion of the *α* into *β* phase, in agreement with the DSC measurement. Finally, the electrical response measured by applying a cyclic strain of 6 Hz generated up to 70% higher voltage per unit force for samples with 7.5–12.5% of ZnO compared to pristine PVDF. High cooling rate printed samples generate an electrical signal up two times large for the same amount of force compared to the LCR specimens. Thus, an overall four times higher voltage per unit force for HCR 10% ZnO-PVDF with respect to LCR pristine PVDF. Furthermore, 10%ZnO-PVDF printed were capable of reproducing a walking profile more accurately than pristine PVDF, and HCR 10% ZnO-PVDF samples also generated a higher volt signal. Thus, suggesting that under the proper printing conditions, the ZnO can be used to promote *β* phase of PVDF nanocomposite material by directly 3D print the desired part without post poling and samples thicker than a thin film. The advantages of the proposed technique for creating parts with PVDF include improved mechanical properties, higher thermal decomposition, increased electrical response and elimination of extra poling steps. Optimization of the process has helped identify the ideal cooling rates and filler content.

## Figures and Tables

**Table 1:** 3D printing parameters.

Parameter	Units	LCR	HCR
Initial layer height	mm	0.27	0.27
Layer height	mm	0.1	0.1
Line width	mm	0.35	0.35
Infill direction	°	[0, 0] & [45, 135]	
Infill percentage	%	100	100
Printing temperature	°C	250	225
Build plate temperature	°C	100	50
Printing speed	mm s^−1^	20	20
Cooling fan	%	0	100

## Data Availability

Additional data will be made available on reasonable request to the authors.

## Supplementary Materials

The following supporting information can be downloaded at: https://www.mdpi.com/article/10.3390/polym14204312/s1.
