# Supplementary material for: Improved Electrical Signal of Non-Poled 3D Printed Zinc Oxide-Polyvinylidene Fluoride Nanocomposites
Source: Polymers (Basel). 2022 Oct 13;14(20):4312. doi: 10.3390/polym14204312 (PMC9610290; doi:10.3390/polym14204312)
Supplement: Supplementary file 1 [file polymers-14-04312-s001.zip › polymers_pvdfznoSI.pdf]

# Supplementary Materials: Improved Electrical Signal of Non-Poled 3D Printed Zinc Oxide-Polyvinylidene Fluoride Nanocomposites

**Sharmad Joshi [1], Enrique Gazmin [2], Jayden Glover [2], Nathan Weeks [2], Fazeel Khan [1], Scott Iacono [2] and Giancarlo Corti [1,*]**

## Supporting Information

**Table S1.** ZnO concentration measurements by weight

| Sample | ave | stdev | error |
|---|---|---|---|
| PVDF | 0 | 0 | 0. |
| 2.5ZnO | 3.093 | 0.113 | 0.06 |
| 5ZnO | 4.683 | 0.213 | 0.12 |
| 7.5ZnO | 6.853 | 0.277 | 0.16 |
| 10ZnO | 9.193 | 1.137 | 0.65 |
| 12.5ZnO | 11.856 | 0.8452 | 0.48 |
| 15ZnO | 14.666 | 0.786 | 0.45 |
| 20ZnO | 20.103 | 0.436 | 0.25 |

avg=average, stdev=standard deviation

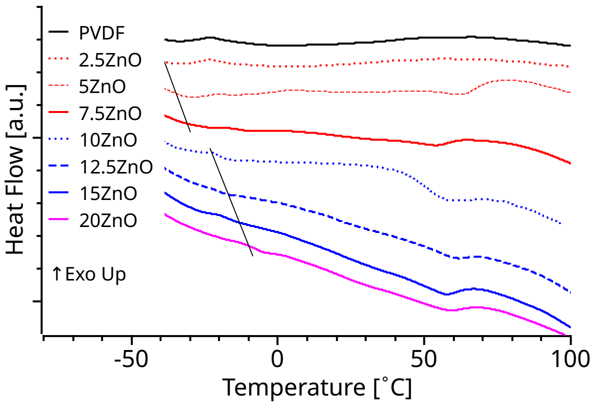


**Figure S1.** Glass transition temperature as function percentage of ZnO content.

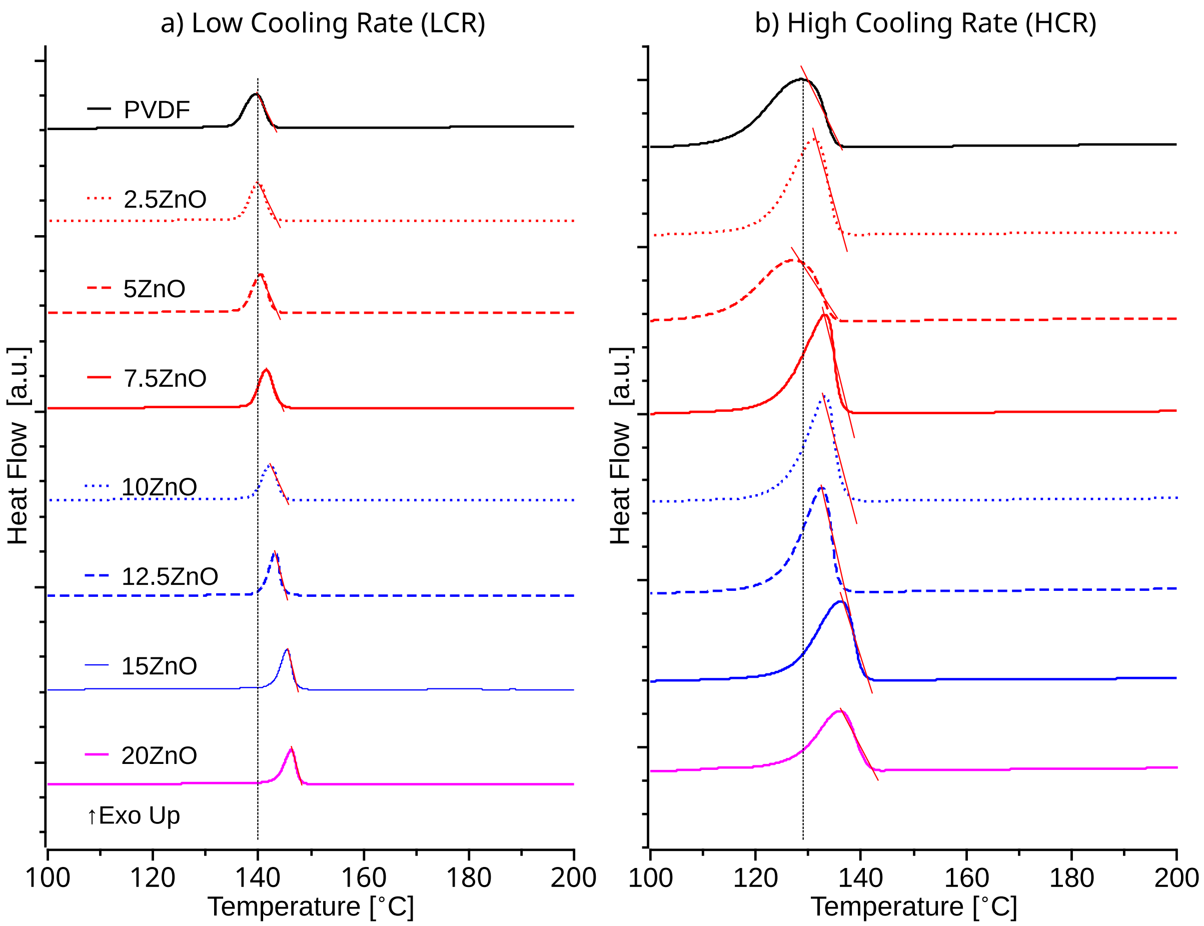


**Figure S2.** DSC crystallization for a cooling rate of a) 5°C $min^{-1}$ LCR, and b) 30°C $min^{-1}$ HCR.

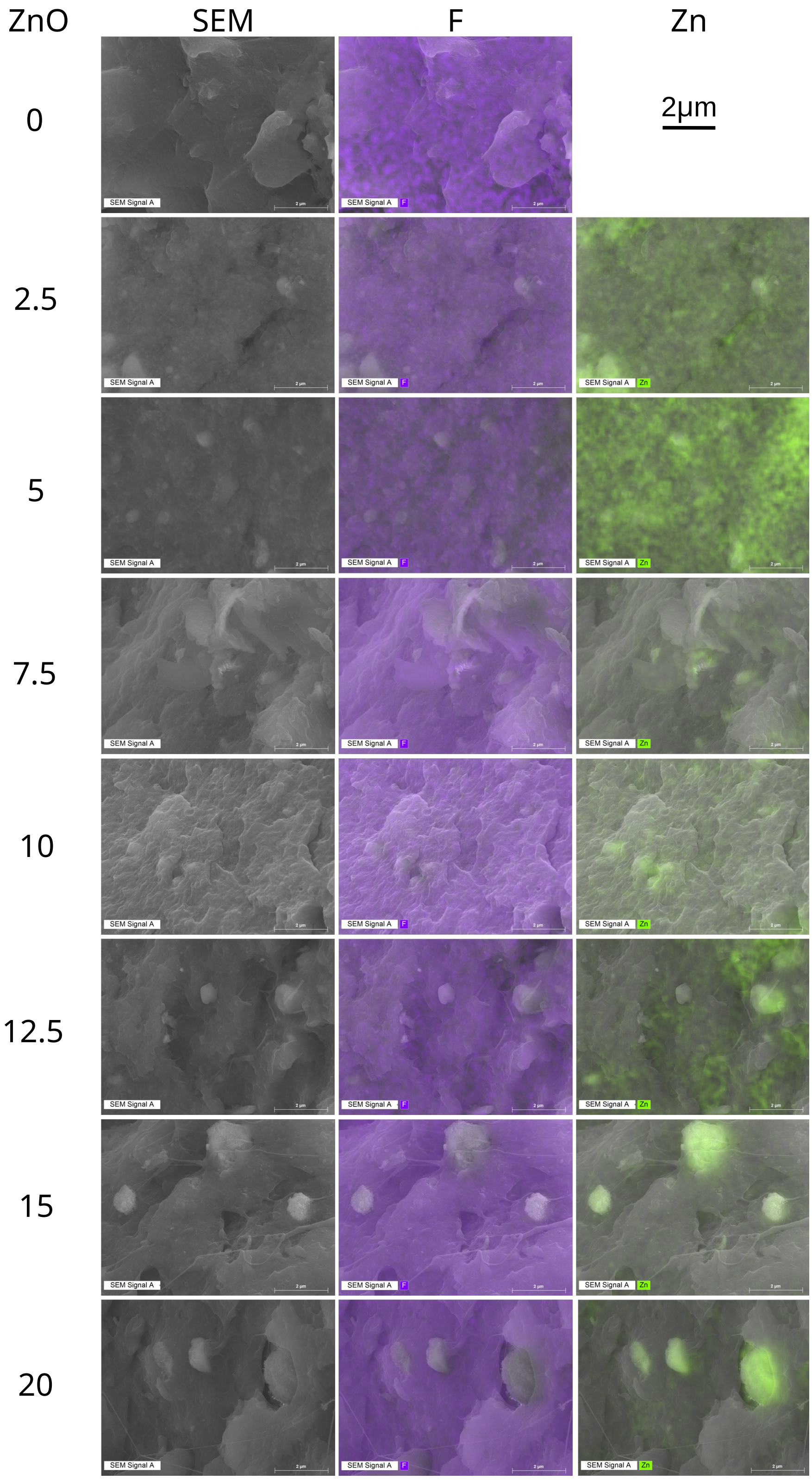


**Figure S3.** XEDS analysis at the failure of 3D printed specimens [45°,135°] tested under tensile stress.

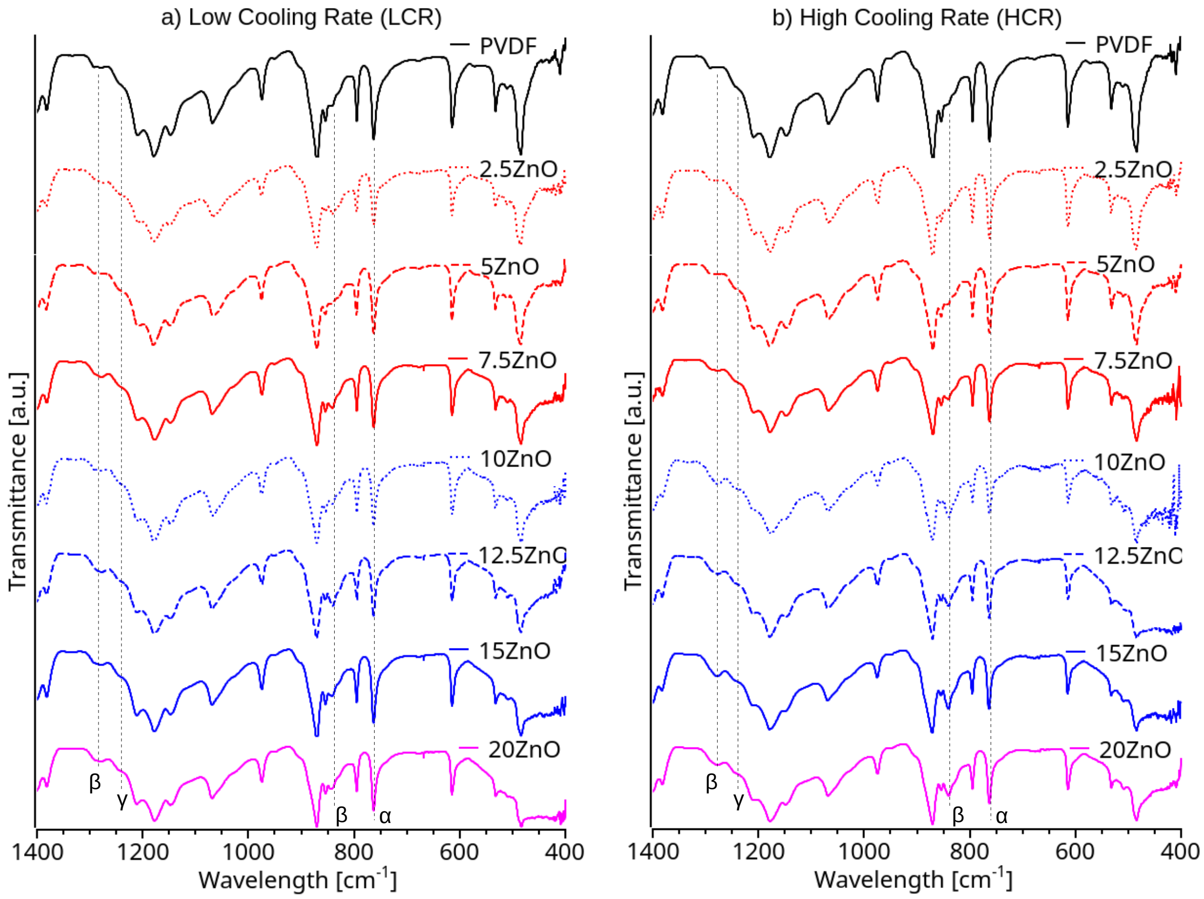


**Figure S4.** FTIR spectra of DSC samples after a cooling rate of: a) 5 °C min$^{-1}$ LCR, and b) 30 °C min$^{-1}$ HCR

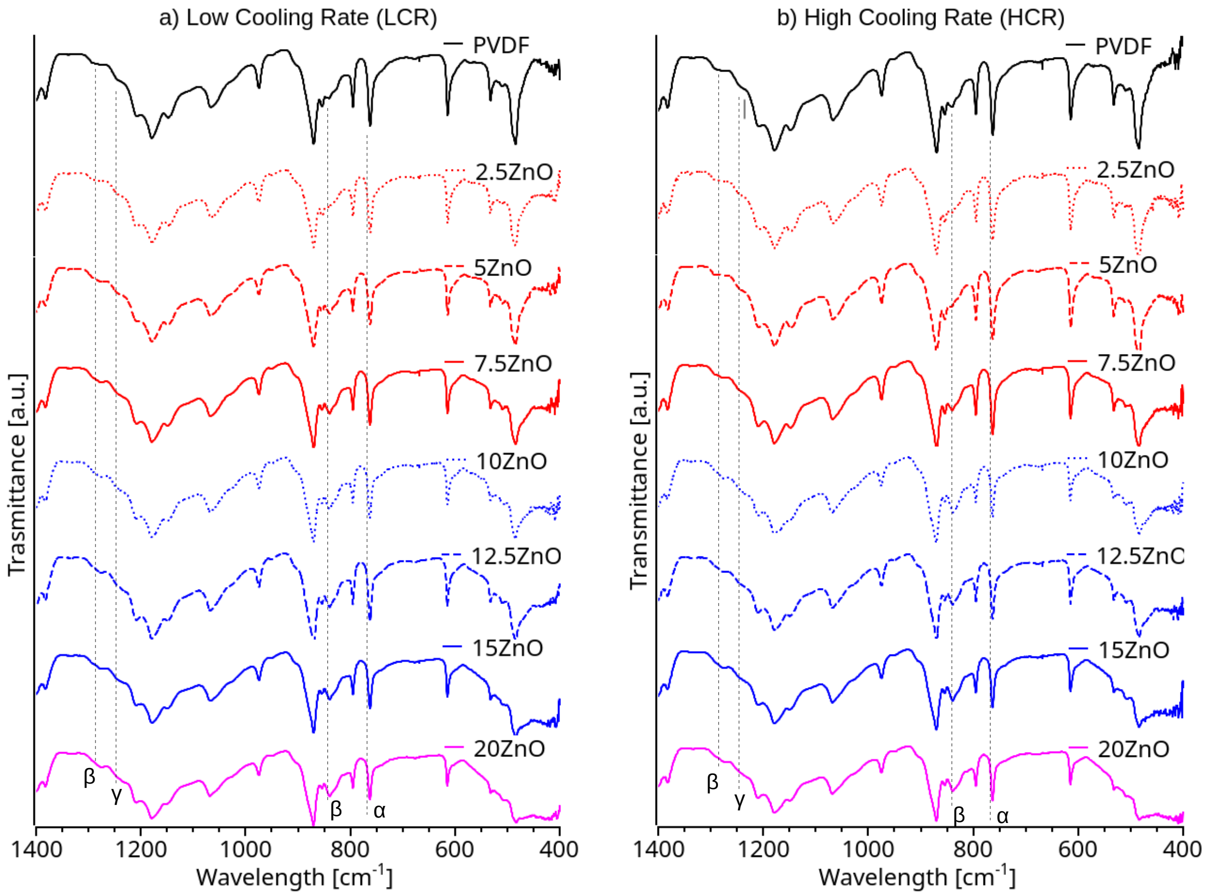


**Figure S5.** FTIR spectra from the top of a 3D printed DMA samples printed with a: a) 5 °C $min^{-1}$ LCR, and b) 30 °C $min^{-1}$ HCR.

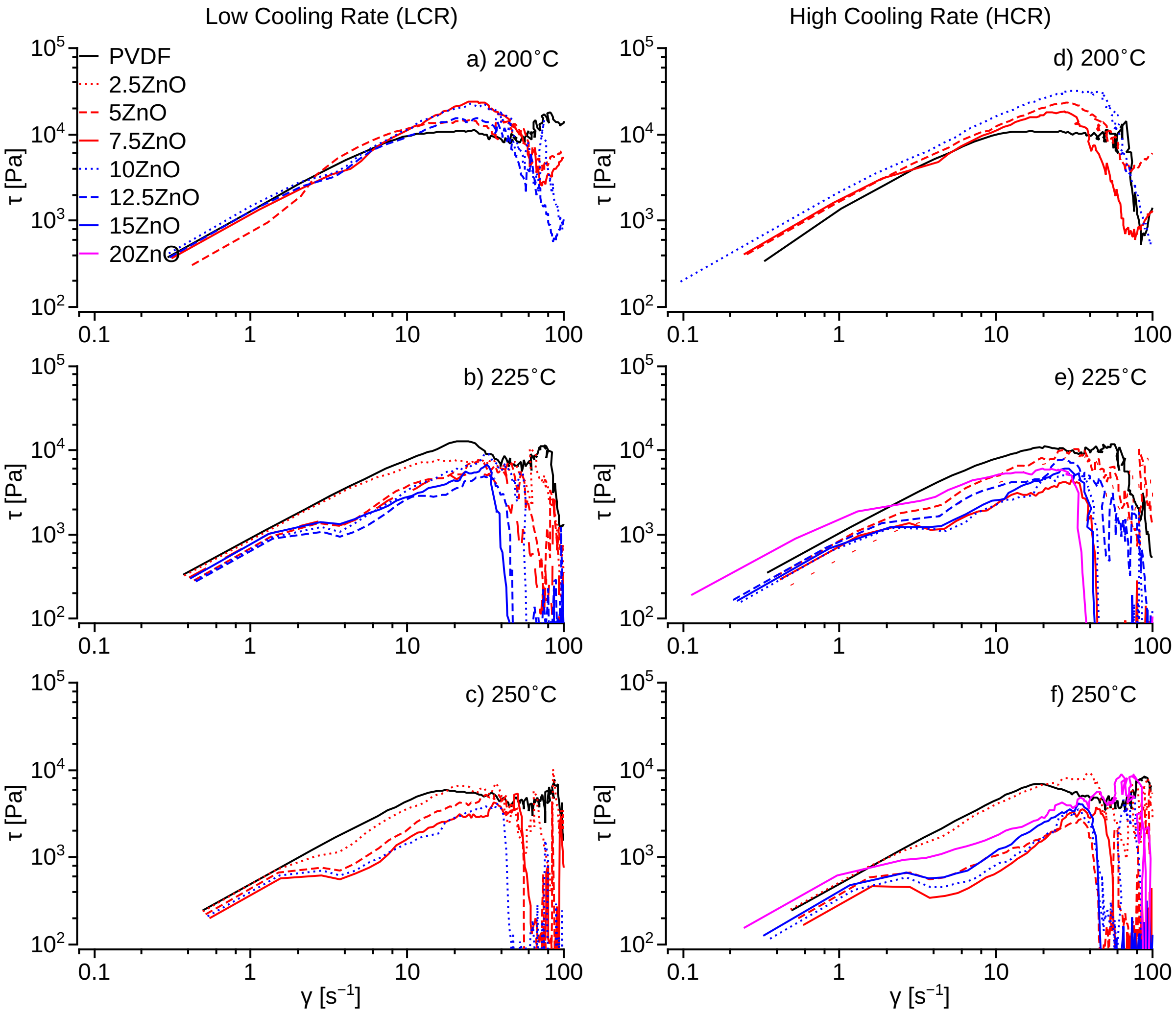


**Figure S6.** Shear stress $\tau$ versus shear rate $\gamma$ at different temperature profiles for low and high cooling rate 3D printed samples.

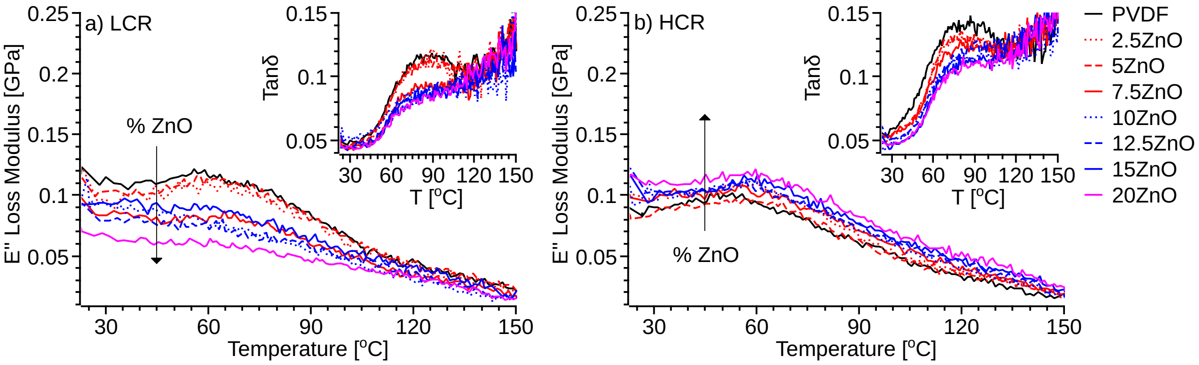


**Figure S7.** Loss modulus (E") of DMA specimens 3D printed with: a) low cooling rate (LCR), and b) high cooling rate (HCR).

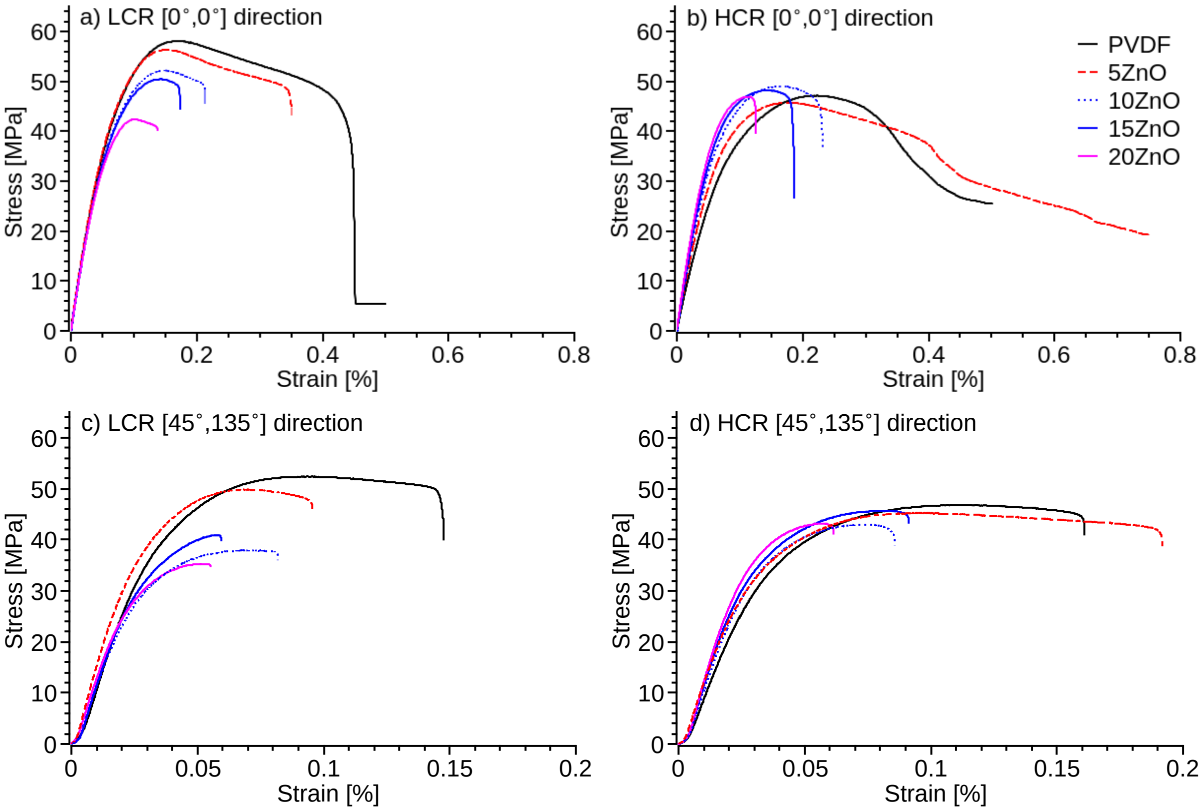


**Figure S8.** Sample stress vs. strain curves of tensile tests for 3D printed a) LCR with infill in the [0°,0°] direction, HCR with infill in the [0°,0°] direction, c) LCR with infill in the [45°,135°] direction, and HCR with infill in the [45°,135°] direction.

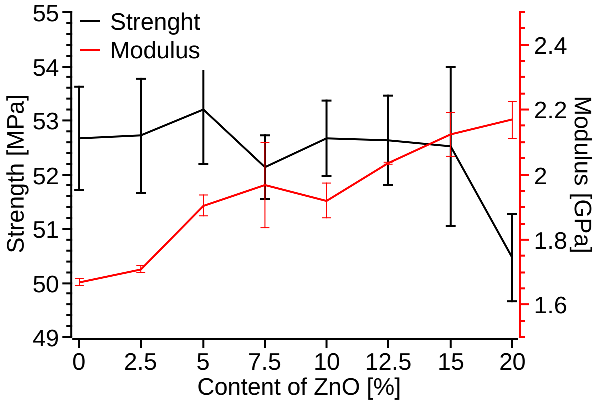


**Figure S9.** Strenght (black) and elastic modulus (red) of the extruded filaments.

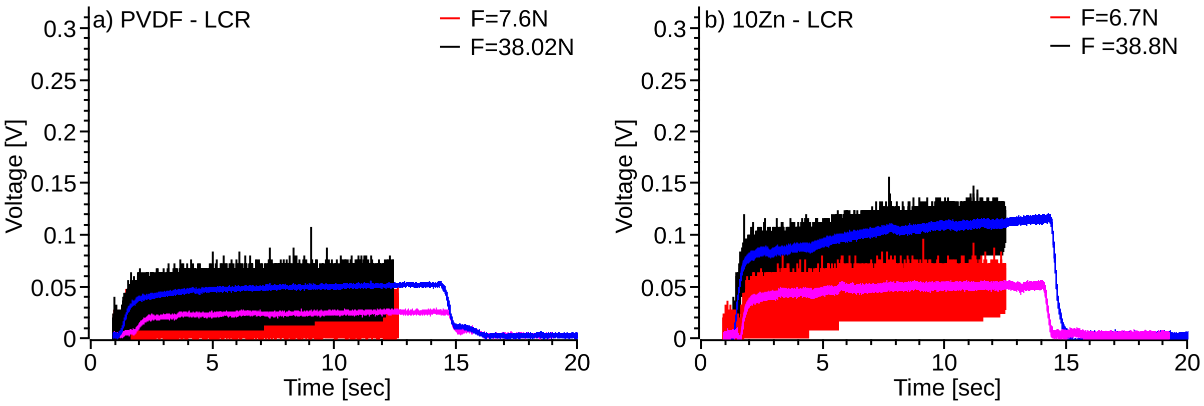


**Figure S10.** Voltage output in response to a 6Hz strain ($\epsilon$) with an amplitude of 0.0058 mm·mm$^{-1}$ (red) and 0.0274 mm·mm$^{-1}$ (black) for LCR samples: a) pristine PVDF and b) PVDF with 10% ZnO. The blue and purple line represents a filtered voltage signal.

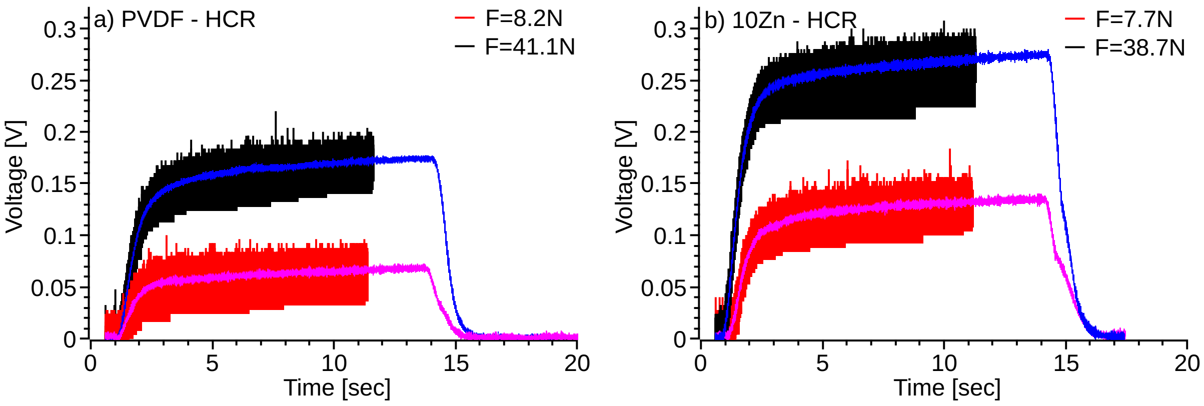


**Figure S11.** Voltage output in response to a 6Hz strain ($\epsilon$) with an amplitude of 0.0058 mm·mm$^{-1}$ (red) and 0.0274 mm·mm$^{-1}$ (black) for HCR samples: a) pristine PVDF and b) PVDF with 10% ZnO. The blue and purple line represents a filtered voltage signal.

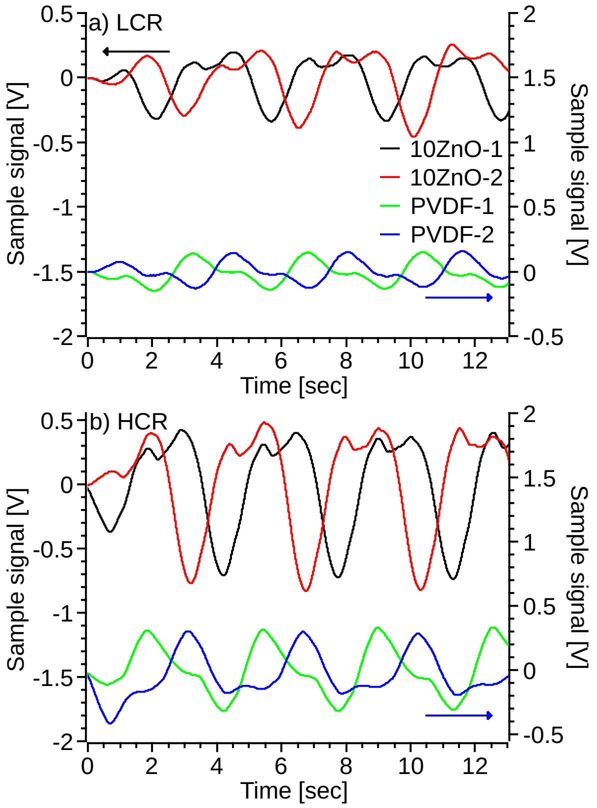


**Figure S12.** Voltage signal generated in response to a walking profile simulated on an MTS tensile frame by two diferent spcimens of pristine PVDF and 10%ZnO PVDF nanocomposite.
